# Readthrough isoform of aquaporin-4 (AQP4) as a therapeutic target for Alzheimer’s disease and other proteinopathies

**DOI:** 10.1186/s13195-023-01318-2

**Published:** 2023-10-11

**Authors:** Pablo Mohaupt, Jérôme Vialaret, Christophe Hirtz, Sylvain Lehmann

**Affiliations:** LBPC-PPC, Université de Montpellier, IRMB CHU de Montpellier, INM INSERM, Montpellier, France

**Keywords:** Aquaporin-4, Waste clearance, Proteinopathies, Alzheimer’s, Protein aggregation, Therapeutic target

## Abstract

The glymphatic system is a crucial component in preserving brain homeostasis by facilitating waste clearance from the central nervous system (CNS). Aquaporin-4 (AQP4) water channels facilitate the continuous interchange between cerebrospinal fluid and brain interstitial fluid by convective flow movement. This flow is responsible for guiding proteins and metabolites away from the CNS. Proteinopathies are neurological conditions characterized by the accumulation of aggregated proteins or peptides in the brain. In Alzheimer’s disease (AD), the deposition of amyloid-β (Aβ) peptides causes the formation of senile plaques. This accumulation has been hypothesized to be a result of the imbalance between Aβ production and clearance. Recent studies have shown that an extended form of AQP4 increases Aβ clearance from the brain. In this mini-review, we present a summary of these findings and explore the potential for future therapeutic strategies aiming to boost waste clearance in AD.

## Background

Translation is a critical biological process that entails the conversion of genetic information encoded in messenger RNA (mRNA) into functional proteins. Traditionally, eukaryotic mRNA was thought to be inherently monocistronic, denoting that each mRNA molecule codes for a single protein sequence. Recent advances in ribosome sequencing and proteomics research have challenged the traditional view of protein translation, revealing a more complex translational landscape [1–4]. It is notable that assumptions regarding protein translation are deeply ingrained in proteomics research, as protein databases are built upon these assumptions and do not encompass the entirety of the human proteome. We recently reviewed microproteins and dual-coding properties of mRNA in neurobiology and their potential involvement in neurodegenerative disorders [5]. For example, transcripts coding for prion protein (PrP), A2A adenosine receptor (A2AR), ataxin-1, and FUS encode an additional protein besides the reference protein [6–9]. Notably, the alternatively translated proteins can serve distinct functions. For instance, two proteins translated from the same FUS mRNA have distinct functions but also both contribute to FUS-mediated motor neuron toxicity [9]. The aforementioned proteins have distinct amino acid sequences due to translation in a different reading frame or in untranslated regions of mRNA. A better-known phenomenon of diversity at the protein level is protein isoforms. Protein isoforms are typically defined as proteins translated from alternatively spliced transcripts. However, shorter or longer versions of a protein with distinct functionality can also be translated from the same transcript. For instance, a shorter version of the protein peripherin is co-expressed with the reference protein, due to in-frame translation initiation occurring downstream of the presumed start codon [10]. This isoform is involved in the process of filament network formation. Recent studies describe the discovery of an extended version of the protein aquaporin-4 (AQP4X), expressed in rat, mice, and human [11, 12]. The translation initiation occurs at the same start codon as the reference protein aquaporin-4 (AQP4), but in some occasions, the elongation does not stop at the stop codon resulting in an extended version of the protein. Hence, this isoform is not formed due to polycistronic features of the DNA or mRNA but due to regulatory aspects of protein translation.

## Amyloid-beta clearance via the glymphatic system

**Fig. 1:**
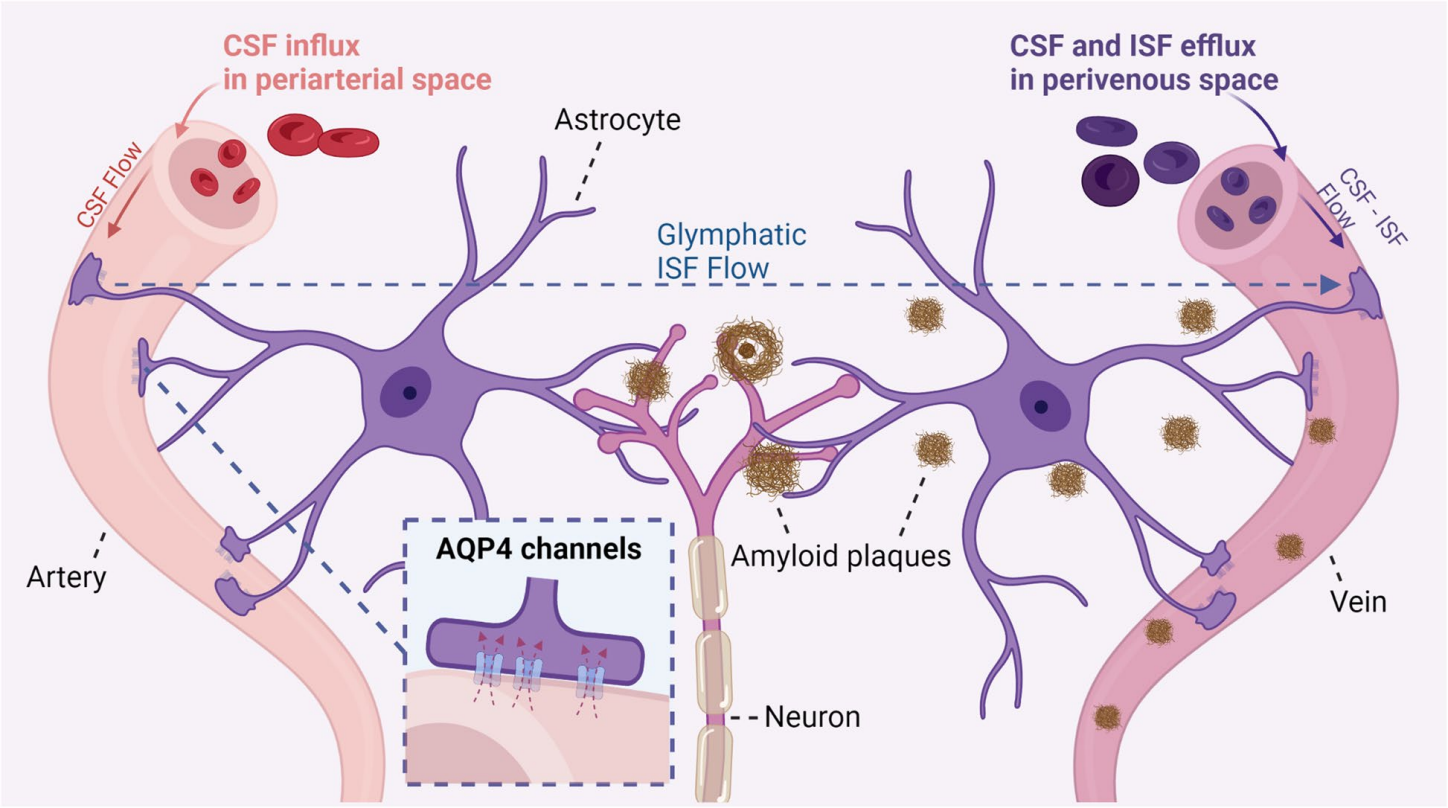
Amyloid-beta clearance via the glymphatic system. Aquaporin-4 (AQP4) water channels facilitate the continuous interchange between cerebrospinal fluid and brain interstitial fluid by convective flow movement. These water channels are positioned on the endfeet of astrocytes situated at the outer layer of the artery and vein. The glymphatic flow guides proteins, such as amyloid-beta, and metabolites to the perivenous space via AQP4 water channels, thus promoting their efficient elimination from the central nervous system outside the brain

Alzheimer’s disease (AD) is characterized by amyloid-beta (Aβ) deposits and hyperphosphorylated tau tangles within the brain. To maintain brain homeostasis, various clearance systems exist to eliminate extracellular Aβ from the central nervous system, including the glymphatic pathway [13]. AQP4, which is located perivascular on astrocyte endfeet, plays a vital role in proper functioning of the glymphatic system (Fig. 1) [14].

Studies using infused radiolabeled Aβ_1-40_ indicate that AQP4-knockout mice exhibit a 55% lower Aβ clearance rate than wild-type mice [15]. Moreover, in an amyloid-beta precursor protein/presenilin 1 (APP/PS1) transgenic mouse model for AD, AQP4-knockout mice show a reduced Aβ clearance rate of 25–50% compared to transgenic mice without AQP4-knockout [16]. The AQP4 levels in cerebrospinal fluid are higher in neurodegenerative dementia, but the perivascular localization of AQP4, which is elemental to proper functioning of the glymphatic system, is disrupted in AD [17, 18]. Several studies indicate that the isoforms exhibit divergent subcellular localizations: the AQP4X isoform is predominantly associated with astrocytic endfeet proximate to vasculature, whereas the canonical AQP4 is situated in the parenchyma, distanced from the vascular structures [11, 12]. This suggests that the translation of the extended isoform could potentially improve waste clearance from the CNS. AQP4X is translated inherently, but the stoichiometry between AQP4 and AQP4X is disrupted in gliosis and, more specifically, the AQP4X/AQP4 ratio was found to be decreased in AD in APP/PS1 transgenic mice models [11, 19]. In an effort to increase the readthrough rate using apigenin and sulphaquinoxaline, and thus increase the translation of AQP4X, an increase in Aβ clearance was observed [19]. These findings suggest that promoting waste clearance could be a valuable tool for slowing down disease progression in AD. Further research is necessary to determine the clinical potential of this approach in long term studies evaluating plaque formation and cognitive function. The application of clustered regularly interspaced short palindromic repeats (CRISPR) and CRISPR-associated protein 9 (Cas9) gene-editing technology presents a potential alternative strategy for future implementation [20]. However, it should be noted that this approach may result in the complete cessation of the translation of canonical AQP4, while both isoforms might be required for maintaining brain health. Additionally, it would be valuable to evaluate if the clearance is specific for Aβ or if this strategy also has potential for other proteinopathies. For instance, α-synuclein build-up is associated with synucleinopathies such as Parkinson’s disease (PD), dementia with Lewy bodies (DLB), and multiple system atrophy (MSA), but accumulating evidence also suggests its involvement in the pathophysiology of AD [21]. To evaluate this paradigm would particularly be of interest since the decreased expression of AQP4 in a PD mice model showed accelerated deposition of α-synuclein [22, 23].

Therapeutic strategies for AD, such as aducanumab and lecanemab, rely on antibodies that target Aβ or Aβ protofibrils. Furthermore, there has been recent interest in microglial activation as a therapeutic strategy to slow down Aβ accumulation and tau deposition [24]. Aβ-targeting antibodies have shown potential in neutralizing Aβ, suggesting a path for enhanced clearance. Once activated, microglia appear primed to efficiently phagocytose and degrade these antibody-tagged aggregates [25]. Enhancing waste clearance mechanisms, such as the glymphatic system, might not only slow the amyloid aggregation process but might also aid in the effective spread of antibodies within the brain tissue due to improved glymphatic flow. The potential synergy between waste clearance mechanisms and Aβ-targeting antibodies remains an intriguing avenue for future therapeutic strategies in AD.

## Conclusions

The enhancement of waste clearance from the brain via the glymphatic system is a novel therapeutic approach for AD. While the definitive clinical implications of AQP4, AQP4X, and the glymphatic system are yet to be ascertained, this avenue suggests a potential direction for future therapeutic exploration. These findings also suggest that promoting waste clearance holds promise for slowing down protein accumulation in other proteinopathies.

## Data Availability

Data sharing is not applicable to this article as no datasets were generated or analyzed during the current study.

## Abbreviations

A2AR: A2A Adenosine receptor
Aβ: Amyloid-β
AD: Alzheimer’s disease
APP: Amyloid-beta precursor protein
AQP4: Aquaporin-4
AQP4X: Extended aquaporin-4
CNS: Central nervous system
CRISPR/Cas9: Clustered regularly interspaced short palindromic repeats and CRISPR-associated protein 9
DLB: Dementia with Lewy bodies
DNA: Deoxyribonucleic acid
mRNA: Messenger RNA
MSA: Multiple system atrophy
PD: Parkinson’s disease
PrP: Prion protein
PS1: Presenilin 1
